# Identification of rare copy number variations reveals *PJA2*, *APCS*, *SYNPO*, and *TAC1* as novel candidate genes in Autism Spectrum Disorders

**DOI:** 10.1002/mgg3.786

**Published:** 2019-06-29

**Authors:** Tania Bitar, Walid Hleihel, Sylviane Marouillat, Sandrine Vonwill, Marie‐Laure Vuillaume, Michel Soufia, Patrick Vourc'h, Frederic Laumonnier, Christian R. Andres

**Affiliations:** ^1^ INSERM U1253 ibrain Université de Tours Tours France; ^2^ Faculty of Sciences Holy Spirit University of Kaslik (USEK) Jounieh Lebanon; ^3^ CHRU de Tours Tours France; ^4^ Faculty of Medicine Holy spirit University of Kaslik (USEK) Jounieh Lebanon

**Keywords:** *APCS*, Autism Spectrum Disorders, CGHarray, copy number variations, *PJA2*, *SYNPO*, *TAC1*

## Abstract

**Background:**

There is a strong evidence for genetic factors as the main causes of Autism Spectrum Disorders (ASD). To date, hundreds of genes have been identified either by copy number variations (CNVs) and/or single nucleotide variations. However, despite all the findings, the genetics of these disorders have not been totally explored.

**Methods:**

Thus, the aim of our work was to identify rare CNVs and genes present in these regions in ASD children, using a high‐resolution comparative genomic hybridization technique and quantitative PCR (qPCR) approach.

**Results:**

Our results have shown 60–70 chromosomal aberrations per patient. We have initially selected 66 CNVs that have been further assessed using qPCR. Finally, we have validated 22 CNVs including 11 deletions and 11 duplications. Ten CNVs are de novo, 11 are inherited and one of unknown origin of transmission. Among the CNVs detected, novel ASD candidate genes *PJA2*, *SYNPO*, *APCS,* and *TAC1* have been identified in our group of Lebanese patients. In addition, previously described CNVs have been identified containing genes such as *SHANK3*, *MBP*, *CHL1*, and others.

**Conclusion:**

Our study broadens the population spectrum of studied ASD patients and adds new candidates at the list of genes contributing to these disorders.

## 1 INTRODUCTION

Autism Spectrum Disorders (ASD) are defined as complex neurodevelopmental disorders with genetic and environmental causal factors. The heritability in ASD is estimated between 51% and 80%, suggesting that the genetic background plays an important role in the pathogenesis of ASD (Pettersson, Lichtenstein, Larsson, & Song, 2019) (Chen et al., 2017). However, the genetic etiology of these disorders remains very complex. It has been shown that single nucleotide variants (SNVs), rare copy number variations (CNVs), and chromosomal abnormalities are associated with ASD (Hnoonual et al., 2017). Rare genomic CNVs, observed in 15%–20% of patients (Huguet, Ey, & Bourgeron, 2013), contribute significantly to the genetic architecture of ASD. To date, hundreds of CNVs, involving one or more genes have been identified in several neurodevelopmental disorders including ASD, intellectual disability, and schizophrenia, some of them being implicated in more than one disorder (Guilmatre et al., 2009). For instance, microdeletions and microduplications in 1q21.1, 7q11.23, 15q11‐q13, 16p11.2, 22q11.2, and 22q13.3 regions (Sanders et al., 2015) have been repeatedly reported in various studies on ASD. Several studies suggested that de novo mutations account for about 16% of the genetic abnormalities detected in ASD (Iossifov et al., 2012). Among the genes mutated in ASD, a significant proportion includes genes encoding proteins involved in synaptic function, ubiquitination and chromatin remodeling.

Taking the evidence that the genetic causes of ASD are not totally elucidated, further studies to identify known and novel genes related to ASD should help in genetic counseling, in the evaluation of recurrence risk in the families and for a better understanding of the pathophysiology in ASD. Thus, the aim of our study was to evaluate the presence of rare CNVs in a group of ASD Lebanese patients, using high resolution comparative genomic hybridization technique (array‐CGH) and real time PCR (qPCR).

## 2 MATERIAL AND METHODS

### 2.1 Sample collection

Autistic patients were diagnosed and enrolled in the study using Diagnostic and Statistical Manual of Mental Disorders, 4th edition criteria, and Childhood Autism Rating Scale (CARS). The average score on the CARS for the patient's sample was in favor of moderate autism. Most of the participants presented a moderate intellectual disability (Bitar et al., 2018). Patients were selected through specialized institutions and nongovernmental organizations (NGOs) specialized in mental disorders in all the districts of Lebanon. All families and participants provided informed consent. Whole blood samples were collected from 19 patients and their parents (Table 1).

**Table 1:** Characteristics of patients with ASD included in our study

Patient number	Gender	Region	Parents consanguinity	Family history	Additional clinical features
12	Male	Beirut	1st degree cousins	N.A	Hyperactivity, echolalia
23	Male	Beirut	No	Diabetes, cancer, and renal disease in both paternal and maternal side	Hyperactivity, anxiety
34	Male	South	1st degree cousins	N.A	Hyperactivity, echolalia
45	Female	South	No	N.A	Epilepsy, speech delay
51	Male	South	No	N.A	Deafness, hyperactivity, anxiety, echolalia
61	Female	South	1st degree cousins	Asthma in maternal side	Hyperactivity, echolalia, anxiety
64	Male	Mount Lebanon	No	Hypertension and high cholesterol in maternal side	Anxiety
70	Male	Bekaa	2nd degree cousins	Diabetes, hypertension, high cholesterol, and triglycerides in both maternal and paternal family side.	Anxiety, depression, hyperactivity, self‐injurious behavior
73	Male	Bekaa	No	Diabetes, hypertension in both family side. Intellectual disability in paternal side	Anxiety, depression, hyperactivity, self‐injurious behavior, echolalia
76	Male	Bekaa	No	N.A	Epilepsy, fear, deafness, anxiety, intellectual disability
79	Male	Bekaa	No	N.A	Depression, hyperactivity, self‐injurious behavior
82	Male	Bekaa	No	Intellectual disability in maternal side/ growth retardation siblings	Deafness, hyperactivity, anxiety, echolalia,, developmental delay, delays in language, motor, and social development.
85	Male	Bekaa	No	N.A	Deafness, hyperactivity, anxiety, echolalia, depression,
88	Male	Bekaa	No	Diabetes in maternal side/ Hypertension in both family sides	Depression, fear, anxiety, self‐injurious behavior, bedwetting
91	Male	Bekaa	1st degree cousins	Diabetes in both sides/ Hypertension, high cholesterol and cardiac disease in paternal family side	Deafness, epilepsy, fear, anxiety, self‐injurious behavior
92	Male	Bekaa	No	Hypertension in maternal family side	Down syndrome, echolalia, hyperactivity, fear, anxiety, self‐injurious behavior
110	Female	North	1st degree cousins	Hypertension in both family side	Deafness, echolalia, hyperactivity, self‐injurious behavior
114	Male	North	No	N.A	Deafness, depression, hyperactivity, fear, anxiety
117	Male	North	No	N.A	Deafness

### 2.2 Array‐CGH investigation

Genomic DNA was extracted from patients with ASD and their parents using the QIAsymphony robot and then measured by spectrophotometry (Thermo Scientific Nanodrop 2000) in order to assess the concentration and purity of the DNA. For CNV detection, we used the high resolution Agilent 2*400K chip consisting of 400,000 60‐mer oligonucleotide probes (SurePrint G3 Human CGH Microarray Kit, 2x400K) with 5.3Kb overall median probe spacing. We have followed the protocol of preparation given by Agilent Company (Agilent Oligonucleotide Array‐Baser CGH for Genomic DNA Analysis, Version 7.5, June 2016).

### 2.3 Protocol

One microgram of genomic patient DNA and a reference sample, provided by Agilent, of the same sex were first digested with the restriction enzymes AluI and RsaI for 2 hr at 37°C. Enzymes were then inactivated at 65°C for 20 min. Digested test and reference DNA (between 200 and 500 base pairs in length) were then labeled with differentially fluorescent nucleotides (the test sample is labeled with cyanine 5 and the reference with cyanine 3) using random primers and exo (‐) Klenow for 2 hr at 37°C. Enzymes were then inactivated at 65°C for 10 min. Labeled DNA were then purified and mixed in the presence of human cot‐1 DNA. Samples were hybridized into SurePrint G3 Human CGH Microarray Kit, 2x400K at 65°C for 40 hr. After microarray washing step, the chip was scanned using SureScan Microarray Scanner (Agilent Technologies) generating an image.

### 2.4 Data analysis

The analysis of the image was processed using Agilent CytoGenomics software 3.0.6.6. Raw image was extracted using Feature Extraction. Quality control was performed and only samples having a derivative log ratio spread <0.25 were kept for further analysis. For CNV detection, we used the Aberration Detection Method‐2 developed by Agilent Technologies which uses derivative log(2) ratio to detect any change in copy number state. A minimum of three probes was used as a threshold for a positive call for an aberration. The threshold was set very low in order not to miss any potential aberrations. We obtained a file containing all the structural variations detected and a graphical representation of each probe in the genome. We applied several filters in order to select the CNVs. At first, we excluded the CNVs observed in the reference. The remaining CNVs were then analyzed using UCSC genome browser (https://genome.ucsc.edu/) in order to sort them into intergenic and intragenic rearrangements. We only kept in our study the intragenic CNVs. We used the Database of Genomic Variants (DGV) to keep only the CNV with a frequency of less than 1% in the general population (Pinto et al., 2010). The genes included in these candidate regions were then analyzed using Cartagenia database that combines several databases including DGV, Decipher, ClinGen. CNVs that were found related to ASD in several databases were classified as pathogenic. CNVs reported in some cases of ASD without a direct evidence of pathogenicity were classified as likely pathogenic, and those without any reported information were classified as unknown significance. The candidate CNVs were then analyzed by qPCR in order to validate their presence and the mode of transmission. Furthermore, Pathway Studio software (https://mammal.pathwaystudio.com/) was used in order to identify the biological functions associated with the novel genes (*PJA2‐SYNPO‐APCS‐ TAC1*) identified in our study (Figure 2). These genes were integrated in the software and the cell process within the network builder allowed us to visualize the biological function network.

### 2.5 Real time PCR

Quantitative real‐time PCR reactions were performed in duplicate in 96‐well plates containing 10 ng of DNA, 5 µM of each primer and 5 µl of Ultra‐Fast SYBR® Green QPCR Master Mix (Agilent Technologie, Santa Clara, CA). The choice of primers for the amplifications of the genes was carried out using the Primer3Plus software (http://www.bioinformatics.nl/cgi-bin/primer3plus/primer3plus.cgi), then the specificity of each amplicon was controlled by the BLAST program (http://blast.ncbi.nlm.nih.gov/Blast.cgi). Each amplicon had a size between 100 and 200 bp. The qPCR reaction was performed using the Light Cycler® 480 (Roche Diagnostics, Basel, Switzerland). For each candidate gene, the experiment was done at least two times. In order to assess the variation of the amplification, the average of the experiments and normalization to one of the parents were performed. The threshold values were less than 0.7 for deletion and more then 1.25 for duplication (Prasad et al., 2012). The primer sequences of the validated genes are shown in supplementary data.

## 3 RESULTS

**Figure 1:**
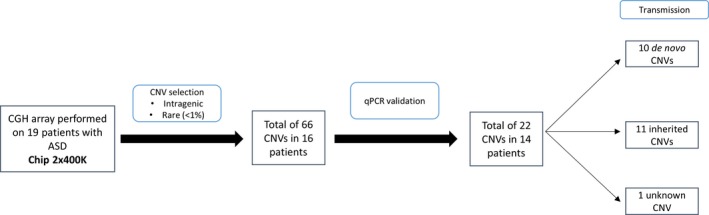
Synthesis of the results obtained by microarray CGH and qPCR

A high resolution microarray CGH was performed for each patient to detect copy number variants. The synthesis of the results obtained by microarray CGH is shown in Figure 1.

We initially detected 60–70 chromosomal aberrations per patient. Then, we applied several filters in order to select the candidate CNVs. We selected 66 CNVs in 16 patients that we have further assessed using qPCR. Thus, we have validated the presence of 22 CNVs (33.3%) in 14 patients including 11 deletions and 11 duplications (Table 2). Among the 22 CNVs validated by qPCR, 10 are de novo (45.4%), 11 are inherited (50.0%) and 1 of unknown origin of transmission (4.5%) because of the death of one of the parents.

**Table 2:** Summary of the CNVs detected in 14 patients

Patient	Sex	Type	Chr	Cytobande	Start	stop	Size (kb)	Genes	Inherited/ de novo	Classification
N°88	M	Dup	1	q23.2	159 409 970	159 697 812	287	*OR10J1, OR10J5, APCS, CRP*	de novo	Unknown significance
		Del	20	p13.1	2 178 080	2 347 300	169	*TGM3‐LOC388780*	Father	Unknown significance
N°76	M	Del	3	q26.31	175 113 349	175 273 066	159	*NAALADL2, MIR548AY*	Father	Unknown significance
N°92	M	Dup	3	p26.3	159 711	746 842	587	*CHL1, CHL1‐AS1, LINCO1266*	Mother	Likely pathogenic
		Dup	21	q11.2‐q22.3	15 385 818	48 095 856	32,710	*SOD1*	de novo	Pathogenic
N°12	M	Del	4	q26	119 268 247	119 281 419	13	*PRSS12*	Unknown	Unknown significance
N°79	M	Dup	5	q21.3	108 691 687	108 825 461	133	*PJA2*	de novo	Unknown significance
N°51	M	Del	5	q33.1	149 919 134	149 989 319	70	*NDST1, SNYPO*	de novo	Unknown significance
		Dup	10	q26.3	135 252 327	135 378 761	126	*SYCE1*	Mother	Unknown significance
N°85	M	Del	6	q26	162 662 138	162 867 251	205	*PARK2*	Mother	Pathogenic
N°114	M	Dup	7	p14.3	32 226 519	32 502 593	276	*PDE1C, LOC100130673*	Mother	Unknown significance
N°91	M	Dup	7	q21.3	97 321 309	97 387 078	65	*TAC1*	Father	Unknown significance
		Del	18	q23.2	74 428 610	78 010 032	3,581	*LOC100134655, ZNF236, MBP, GALR1, LINC01029, SALL3,CTDP1,*	de novo* (probable parents translocation)*	Pathogenic
		Dup	22	q13.1‐q13.33	45 172 457	51 224 252	6,051	*SHANK3 and 49 other genes*	de novo (probable parents translocation)	Pathogenic
		Dup	X	q28	148 314 250	148 654 024	339	*IDS, CXorf40A*	Mother	Unknown significance
		Dup	13	q13.31‐q13.33	39 514 479	39 544 503	30	*STOML3*	Mother	Unknown significance
N°117	M	Del	7	p13	44 848 944	44 924 128	75	*PURB, H2AFV*	de novo	Unknown significance
		Dup	16	q23.1	77 176 889	77 273 344	96	*MON1B, SYCE1L*	Mother	Unknown significance
N°34	M	Del	11	q14.1	77 820 274	77 829 284	9	*ALG8*	Mother	Unknown significance
N°110	F	Del	16	p13.1	15 048 676	15 116 406	67	*PDXDC1*	de novo	Unknown significance
N°61	F	Del	16	p13.1	15 048 676	15 116 406	67	*PDXDC1*	de novo	Unknown significance
N°82	M	Del	22	q13.33	51 123 491	51 219 009	95	*SHANK3, LOC105373100, ACR, RPL23AP82, RABL2B*	de novo	Pathogenic

### 3.1 De novo CNVS

Ten de novo CNVs (six deletions and four duplications) were found in nine patients. We found 2 CNVs located in 22q13.33: a de novo deletion of 95 Kb (patient no 82) containing the *SHANK3* (OMIM # 60623) gene (Table 2), and a de novo duplication of 6,051 Kb (patient no 91) comprising 50 genes including the *SHANK3* gene. These CNVs are classified as pathogenic as there were clearly found related to ASD in the AutismKB database (http://autismkb.cbi.pku.edu.cn/) and Sfari gene database (https://gene.sfari.org/). Furthermore, a de novo deletion of 3 Mb that encompasses several genes including *MBP* (OMIM #159430) gene was identified in patient no 91 and classified as pathogenic. Both duplication in the region 22q13.33 and deletion in the region 18q23.2 found in patient no 91 are terminal and it is most likely that this represents an unbalanced translocation, potentially inherited from a balanced translocation of one of the parents.

The same de novo deletions of 67 kb were identified in two unrelated female patients (no 61 and 110) at 16p13.1 region involving the *PDXDC1* (OMIM #614244) gene of unknown significance.

### 3.2 *Novel *de novo* candidate regions*

We have detected three potential de novo rearrangements. One de novo duplication of 133 kb in patient no 79 was localized at 5q21.3, that includes the *PJA2* (OMIM #300420) gene. Another de novo deletion of 70 Kb was located at 5q33.1, encompassing two genes including *SYNPO* (OMIM #608155) in patient no 51. Furthermore, a de novo duplication containing the *APCS* (OMIM #104770) gene located at 1q23.2 has been identified.

### 3.3 Inherited CNVs

Eleven inherited CNVs (four deletions and seven duplications) were found in nine patients. Among the CNVs of interest we found a duplication of 587 Kb (patient no 92) located at 3p26.3 that encompasses the *CHL1* (OMIM #607416) gene. We observed in patient no 85 an inherited deletion of 205 Kb located at 6q26 involving the *PARK2* (OMIM #602544) gene, classified as pathogenic. Furthermore, three inherited CNVs were found in patient no 91 in which two duplications were inherited from the mother; one of 30 Kb located in 13q13.31‐q13.33 comprising the *STOML3* (OMIM #608327) gene of unknown significance as there are no reported information, the second of 339 Kb at Xq28 encompassing the *IDS* (OMIM #300823) gene. The third duplication located in 7q21.3 region inherited from the father and involving the *TAC1* (OMIM #162320) gene, has not yet been identified in any study on ASD.

### 3.4 Oligogenic model

For some patients, we have observed at least two candidate CNVs, with one variant inherited from a healthy parent and the other variant inherited from the other healthy parent, or de novo. Our results have shown that four patients were found to have two different CNVs (Two‐hit CNVs) and one patient was found to have five CNVs simultaneously (Table 2).

**Figure 2:**
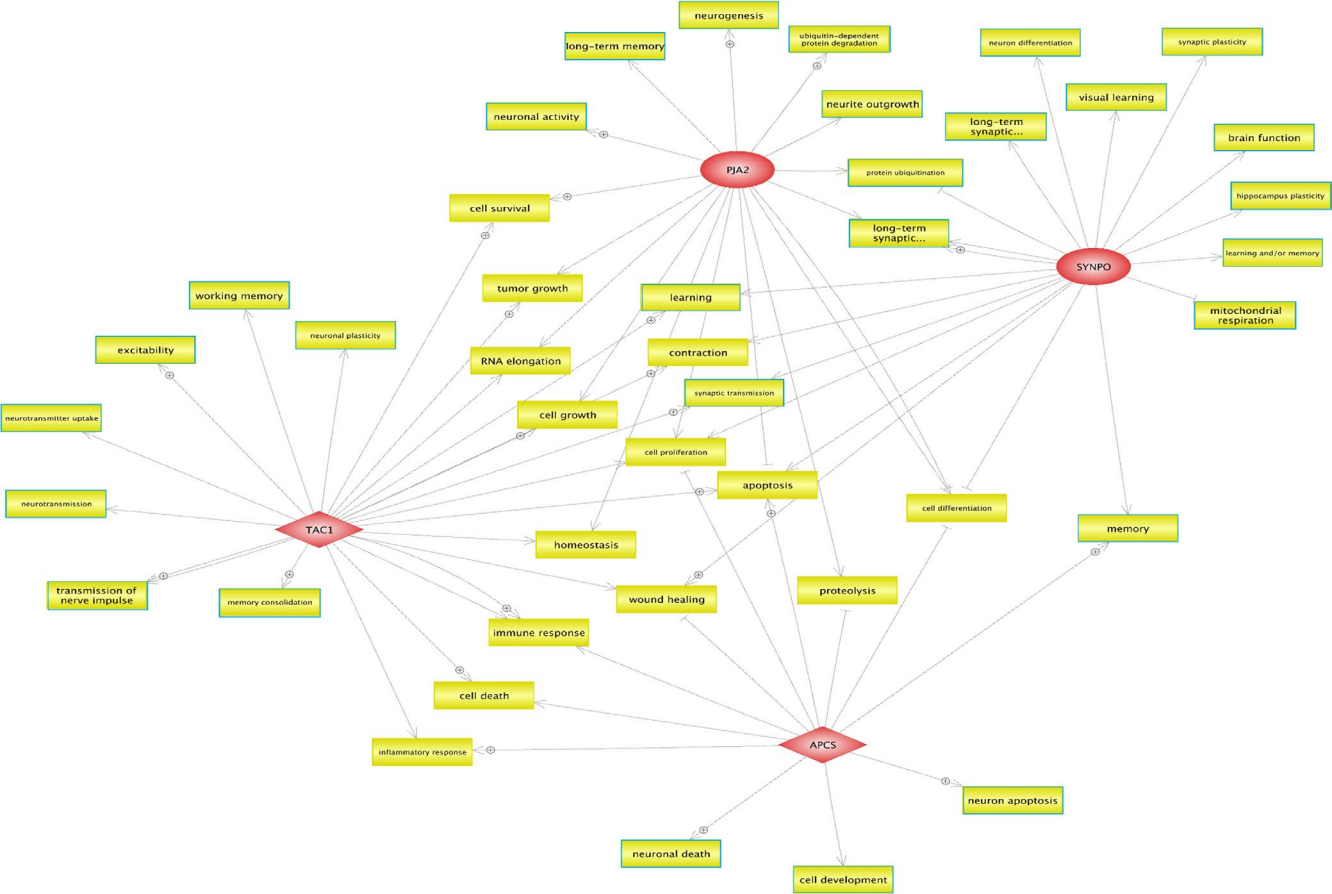
Pathway studio analysis of the four novel candidate genes. A biological network was created connecting genes with their biological functions. Red color represents the novel candidate genes, yellow colors for cellular process and the blue frames represent the functions related to neurodevelopment and synaptic functions

A biological network was created using Pathway Studio program connecting the four identified new candidate genes with their biological functions (Figure 2). We only kept the functions related to neurodevelopment and synaptic activity, as well as those that are in common between genes.

## 4 DISCUSSION

Although ASD are amongst the most heritable neurodevelopmental disorders, their genetic basis is far from being elucidated. It is therefore essential to understand the degree of involvement and causality of the identified genetic variants in this complex disorder in which genetics play an important role. ASD may result from mutations in a large group of genes having roles in various physiological processes such as chromatin remodeling, ubiquitin pathways, translation, metabolism, and synaptic functions (Giovedí, Corradi, Fassio, & Benfenati, 2014). To date, no specific genes linked to ASD have been identified, meaning that the genes mutated in ASD can be found in other neurodevelopmental disorders. Hence, it is important to uncover novel contributory genes in ASD that will provide new insights in the etiology, and to confirm previously described genes in ASD that will support the role of these genes in these disorders. In our study, we worked on identifying CNVs in the genome of Lebanese ASD patients, using high resolution in situ hybridization. The first level of analysis of the variant is the inherited versus de novo character. In fact, when the variant is de novo, the gene can be considered as a good candidate for the disorder. Alternatively, when the variant is inherited from a healthy parent, the question of its level of pathogenicity arises immediately: if the variant contains a gene already known as being involved in ASD or another neurodevelopmental disorder, it can be considered as a probable risk factor. Conversely, if the variant contains a gene without particular implication in a disorder of the central nervous system, its interpretation is much more difficult.

In our study, 26% of the patients (5/19) had more than one candidate CNVs observed, which gives an argument in favor of a multihit genetic model. In fact, each parent transmits to his child a CNV that would have little or no individual effect in the carrier parent, but which, in the case of an association with another CNV, de novo or inherited from the other parent, may contribute to ASD.

In addition, the function of the genes present in the CNV is another criterion for selection, and in the case of ASD, genes with significant neuronal expression are obviously retained.

It is also important to mention that gene duplication is more difficult to interpret than deletion. Indeed, duplication may have either the effect of deletion, may lead to a gain of function, or to a modification of the expression profile of the gene, depending on the specific localization of the borders of the duplication.

The results of our study have shown that only 22 CNVs from the 66 detected were validated by qPCR. Indeed, the high resolution of the chip we used can reveal a number of false positives. The non‐validated CNVs represent small deviations that actually correspond to background noise and not to real anomalies.

In our study, previously described and novel ASD genetic loci have been identified. A novel de novo duplication at 1q23.2 (Table 2) that involves *APCS* (amyloid P component serum) which is member of pentraxin family, has been identified. *APCS* encodes a glycoprotein that is thought to control the degradation of chromatin. It has been also established that this protein binds to apoptotic cells at an early stage, which increases the possibility that it is involved in neuron apoptosis in vivo (Figure 2). The activation of apoptotic death pathways under pathological conditions could lead to developmental disabilities and neuronal apoptosis has been proposed as one of the etiological factors of ASD (Wei, Alberts, & Li, 2014). Furthermore, *APCS* gene seems to play a role in several other cellular processes including memory which strengthens the potential role of this gene in ASD (Figure 2).

In addition, a novel de novo duplication of 133 Kb at 5q21.3 has been observed including a novel candidate gene for ASD, *PJA2* (Praja Ring Finger Ubiquitin Ligase 2), which is involved in protein kinase A (PKA) dependent memory regulation. The *PJA2* gene encodes a protein involved in the pathway of protein ubiquitination. The ubiquitin pathway regulates the turnover of synaptic components, especially those related to learning and plasticity (Glessner et al., 2009).

Moreover, a de novo deletion of 70 Kb at 5q33.1 including two genes, *NDST1* (OMIM #600853) and *SYNPO* has been detected. *NDST1* encodes an enzyme of the heparin sulfate pathway involved in the first steps in the synthesis of heparin sulfate chains (Armstrong et al., 2017). A mutation in this gene has been identified in a girl with developmental delay, ataxia and cranial nerves dysfunction (Armstrong et al., 2017). In addition, a duplication of 54,785 bp in the *NDST1* gene has been found in a Canadian autistic patient (Prasad et al., 2012). On the other hand, *SYNPO* has not been reported in any study on ASD. It encodes a Synaptopodin protein involved in synaptic plasticity and may play a role in actin‐based cell shape and motility. The presence of this gene with *NDST1,* which is already known to be implicated in ASD, weakens however its possible role in this disorder.

We have also found an inherited duplication of 65 kb at 7q21.3 which involves the whole *TAC1* gene. As far as we know, no CNVs in this region were detected in ASD till now. There is only one study in which the association between three SNPs of the *TAC1* gene and autism has been searched. This study showed that no association with three SNPs of the *TAC1* locus and autism could be detected (Marui et al., 2007). The *TAC1* gene encodes four products of the tachykinin peptide hormone family, including substance P and neurokinin A, which are neurotransmitters. These products are known to modulate glutamatergic excitatory synaptic transmission (Marui et al., 2007). Thus, our observation opens the question of a possible role of this *TAC1* gene in ASD.

Using Pathway Studio software, functional processes impacting memory, learning, synaptic functions, neurodevelopment and ubiquitination were highlighted and linked to the four genes, which reinforces the importance of neural and synaptic defects in ASD (Figure 2). In addition, Figure 2 shows that these genes are also connected together by other biological functions including immune response, cell death, cell proliferation, and apoptosis which have also been associated with ASD.

Some of the CNVs that we identified in this study contained genes that have been already reported in the literature and may represent robust markers of this disorder. This includes four CNVs of which two are inherited and two are de novo (Table 2) (3p23.6, 6q26, 18q23.2, 22q13.3) involving genes such as *SHANK3* that plays a role in synapses formation and dendritic spine maturation. *SHANK3* deletions produce a neurodevelopmental disorder (Phelan‐McDermid Syndrome), characterized by global developmental delays, intellectual disability, severe speech delays, and ASD (patient no. 82 in which symptoms observed correspond to several of the signs of Phelan‐McDermid syndrome, see Table 1). In addition, *MBP* gene encodes a major constituent of myelin sheath of oligodendrocytes and Schwann cells and *PARK2* is a ubiquitin‐protein ligase, mutations of which cause autosomal recessive juvenile Parkinson's disease (Yin et al., 2016) and which was reported to be linked to ASD and attention‐deficit hyperactivity disorders (Palumbo et al., 2016; Yin et al., 2016). Interestingly, patient no 92 affected by Down syndrome bears also another abnormality on chromosome 3, containing the *CHL1* gene known for its major role in establishing synapses in the brain. In addition, it has been shown that deletion of chromosome 3p25‐p26 was characterized by poor growth, developmental delay, intellectual disability, ASD, and an unusually small head (Li, Liu, Zhou, Hu, & Xu, 2016). This observation may illustrate that the association of ASD with trisomy 21 is perhaps caused by other abnormalities than only trisomy 21.

In addition, we have identified two maternally inherited duplications, one of 30 Kb at 13q13.31‐q13.33 that encompasses the *STOML3* gene and the other of 339 Kb at Xq28 that includes the *IDS* gene.

STOML3 encodes a protein related to stomatin that controls the activity of ion channels (Tan et al., 2007). Furthermore, stomatin is expressed in receptor neurons of the olfactory epithelium (Nagaishi et al., 2012). Olfactory problems have been reported as predictive factors of social impairment in children with ASD (Kumazaki et al., 2018).

Single nucleotide mutations in the iduronate 2‐sulfatase (*IDS*) gene at Xq28 most commonly cause Hunter syndrome, which can cause developmental delays (Marshall et al., 2013).

In our study, the inherited deletion of 159 Kb at 3q26.31 that involves the N‐acetylated alpha‐linked acidic dipeptidase‐like 2 *NAALADL2* (OMIM #608806) gene has been observed. Our results are concordant with those of a case‐control study in the Taiwanese Han population in which an association was detected between ASD and SNPs within the *NAALADL2* gene (Kuo et al., 2015). *NAALADL2* is implicated in the rare developmental malformation Cornelia de Lange syndrome. Patients with this syndrome are usually characterized by growth retardation, mental disability and distinctive facial features and may present ASD features (Tonkin et al., 2004).

In this work, we have explored the nuclear DNA of ASD patients in a Middle Eastern population by high resolution array‐CGH in order to detect CNVs. Our results have shown that previously described CNVs are present in the DNA of Lebanese ASD patients, meaning that there are common genetic causes between Lebanese ASD and other studied populations. Furthermore, de novo mutations have been identified for the first time. Thus, our findings should be expanded, and functional studies should be performed in order to use the information in the future to improve the understanding and the diagnosis of ASD worldwide.

## CONFLICT OF INTERESTS

The authors declare that they have no conflict of interests.

## ETHICAL APPROVAL

The ethical committee of the higher center of research of USEK (Lebanon) approved the protocol and all parents of participants provided informed consent.
