# Supplementary material for: Identification of rare copy number variations reveals PJA2, APCS, SYNPO, and TAC1 as novel candidate genes in Autism Spectrum Disorders
Source: Mol Genet Genomic Med. 2019 Jun 29;7(8):e786. doi: 10.1002/mgg3.786 (PMC6687626; doi:10.1002/mgg3.786)
Supplement: Supplementary file 1 [file MGG3-7-e786-s001.docx]

**Supplementary data**

**Table S1:** List of the primers sequences of the validated genes

| Patient | Chr | Cytobande | Genes | Primer |
| --- | --- | --- | --- | --- |
| N°88 | 1 | q23.2 | *OR10J1, OR10J5,* ***APCS****, CRP* | ACAGCCACTGTGTTGTCTGC  GCTGGTGAGGACAGAGATCC |
|  | 20 | p13.1 | ***TGM3****-LOC388780* | tcccttgcctcctaggactt  ACCTGGAAGTTTTGGCCTCT |
| N°76 | 3 | q26.31 | ***NAALADL2****, MIR548AY* | TTAGCCTCCACAGTCCCATAAG  agaaaaaggtcggttttgc |
| N°92 | 3 | p26.3 | ***CHL1****, CHL1-AS1, LINCO1266* | ggaaacatactgccaaaccaa  GGAAGAAGACAACCCGGTAA |
|  | 21 | q11.2-q22.3 | ***SOD1*** | GCTGGTTTGCGTCGTAGTCT  AATTGATGATGCCCTGCACT |
| N°12 | 4 | q26 | ***PRSS12*** | GGCAGACCCTGGTGTTTCTA  acacaccccgtcactttttc |
| N°79 | 5 | q21.3 | ***PJA2*** | CCAGCAGGAGGGTATCAGAC  AGCCCGACCTAAGCTTCTTT |
| N°51 | 5 | q33.1 | ***NDST1****, SNYPO* | AAGGATTTTGGTGCCAACTG  aggaaagagaatccccctca |
|  | 10 | q26.3 | ***SYCE1*** | aagcctcccctaaccctgta  actcccacaagcagagagga |
| N°85 | 6 | q26 | ***PARK2*** | ctgcaccccaaatcattctt  tgattgccttgtggtctgag |
| N°114 | 7 | p14.3 | ***PDE1C****, LOC100130673* | gagattgcagggtgagtggt  tcagcttcagcatccattgt |
| N°91 | 7 | q21.3 | ***TAC1*** | gttatgggcatcgacgagtt  AGACCCACGTGACATTCTCC |
|  | 18 | q23.2 | *LOC100134655, ZNF236,* ***MBP****, GALR1, LINC01029, SALL3,CTDP1,* | gggaGGACAACACCTTCAAA  GGAGGGTCTCTTCTGTGACG |
|  | X | q28 | ***IDS****, CXorf40A* | catgagggagggttcagtgt  GCAAAGGCATTCTGGAAGAG |
|  | 13 | q13.31-q13.33 | ***STOML3*** | ctggggagaggggtatcaat  GCTGACTTCAGGGATTTGGA |
| N°117 | 7 | p13 | ***PURB****, H2AFV* | CTGCTGGTGTGCACTCCTTA  cctgattccatacccattcg |
|  | 16 | q23.1 | ***MON1B****, SYCE1L* | ATCCGTTGAAAGGAGTGCAG  ACCCCTCCCAGAAGACACTT |
| N°34 | 11 | q14.1 | ***ALG8*** | TTCCCGACTCTTTCCTTTCA  tcgctaccatacCGATGACA |
| N°110 | 16 | p13.1 | *PDXDC1* | ccttccatccagagtggtgt  aaatgctgggacagacaacc |
| N°82 | 22 | q13.33 | ***SHANK3****, LOC105373100, ACR, RPL23AP82, RABL2B* | CCAGCGATATCAACCTGAAG  TAAAGGGCCACTGATGTTGC |
